# The Making of a Modern Hospital


**Published:** 1911-12-09

**Authors:** 


### THE MAKING OF A MODERN HOSPITAL.*

### VI.—The Ward Unit.

In every modern hospital that fulfils modern requirements we find that the various departments are arranged in a definite system which has been called the "unit or detail system." That is to say, each unit or detail is a complete entity, sufficient by itself to stand alone and to carry on its work, if necessary, without the help of the other departments. At the same time, it is imperative to bear in mind that normally it is never contemplated that such independent working should be the rule. As we have already said, the ideal hospital works harmoniously, each department co-operating with the others. In that respect we may legitimately liken the modern hospital to a great ocean liner like the "Olympic" or the "George Washington," in which there are so many units or departments, each carrying on its special work, in a sense, independently, but as a whole working in complete harmony with the rest. The superintendent of the hospital may be likened to the captain of the ship, who is in supreme control but who has under him expert officers in charge of the various departments, just as the captain has experts in the engine-room, in the purser's department, in the stewards' department, and in the other special units which compose the collective system which he regulates.

### The Old Idea.

The first, and perhaps the most important, unit in hospitals to which we have to pay attention is what is called the ward unit. This consists of the department in which the sick patients are treated, with its appurtenances and adjuncts. In the old type of institution the general ward was an indeterminate arrangement, if this somewhat paradoxical expression may be used in connection with it. A great part of the work it had to do was carried on beyond its actual limits, largely owing to the fact that older hospital architects did not keep in view the fact that compactness was the most essential point in construction. In a modern institution the general ward unit is spoken of as a station (by Continental writers) and as a "unit" by English and American authorities. Such a unit, in order to be complete should contain the following:—

A ward or wards for the patients.

A room for the medical officer on duty.

A room for the nurse in charge of the ward.

A bath room.

Lavatory accommodation for (*a*) the patients, and (*b*) the attendants.

A ward kitchen.

A room for dirty linen, dirty utensils.

A wash closet.

A room for the washing and disinfecting of contaminated utensils.

A day room or verandah for such patients as are not forced to keep their beds.

A ward laboratory or clinical investigation and examining room.

A room for the admission or seclusion of special and temporary cases that require special treatment.

To this list of absolutely necessary complements some foreign authorities add the following:—

An examining room for the admission of patients.

A room for the performance of bad dressings and minor operations.

A room for the moribund patients.

Generally, however, the list as given above is deemed to be complete, and a further extension of the unit is to be deprecated as conducing to unnecessary complication entailing more work on the part of the staff and, therefore, as less economical.

### Rival Theories.

It must not be thought that the complete ward unit as outlined above is a cumbrous arrangement which is highly complex. On the contrary, modern methods of hospital construction, as we shall show in a later article, have designed ward units which for simplicity and compactness may be taken to be unrivalled by the older ward arrangements in which these separate essentials now deemed to be of such primary importance were absolutely disregarded. There is, also, some academical differences of opinion with regard to the exact requirements of the modern ward unit. These we will discuss in more detail hereafter, when we concern ourselves with individual requirements. For the present it is sufficient to state that these differences centre round the question of utility. There is a section of hospital workers who desire to limit the requirements of the unit to what may be called the irreducible minimum. This section, for instance, objects to the addition of the doctors' room, of the nurses' room—in English wards usually called the sisters' room—and the day room, on the ground that these may be dispensed with.

The resident officers, for example, are not constantly in the wards, and can be lodged outside the ward unit; the head nurse, theoretically, at least, has her time so much occupied in the ward that she has no opportunity to use the special room provided for her; while the day room is objected to on the score that the ward is not for convalescent patients, who ought to be sent out to the convalescent homes as soon as possible, but for patients who are using the beds and who do not require a special day-room.

### Three Types of Hospital.

Leaving apart, for the moment, these special objections, which it is easy to understand and apply much more forcibly to the three additions to the complete unit given by some modern authorities, and

* Previous articles in this series appeared in The Hospital of October 7, 14, 21, 28, November 4 and 18.

outlined above, we may deal with the unit as a whole on the assumption that it contains all the complements enumerated in the first list.

First of all, we come to the building in which the unit is situate. For all practical purposes modern hospitals may be divided into three large classes. There are, first, those in which each unit is housed in a separate building, usually of one storey in height, though sometimes more, each such building being known as a "pavilion." This is what is called the pavilion system, and the best examples of this kind of construction are to be found in the Moabite and Virchow hospitals at Berlin, the Eppendorf hospital near Hamburg, the Alexander hospital at St. Petersburg, and the military hospital at Bourges. Depage, Vandervelde, and Cheval, in their recent memoir on hospital construction, enumerate no fewer than twenty-five distinct varieties of the pavilion type. These, however, need not concern us here, although they will have to be more fully discussed when we deal with the methods of hospital planning in a future article. The essentials of the pavilion type are that each unit is housed in a separate building, a group of such buildings or units forming a department.

The second type is where the units are arranged in one or more large pavilions, which are usually of more than one storey in height. This is the "compound pavilion system," which is seen at St. Thomas's Hospital, the new clinics at Munich, the new surgical hospital at Graz, the Leipsig Children's Hospital, the Johns Hopkins at Baltimore, the new Gynæcological Hospital at Vienna, and many other modern hospitals. Its essentials are that each compound pavilion is separate from its neighbour, connected, if there is any connection, only by a corridor. This type economises space, conduces to compactness, and gives several advantages which the single pavilion system lacks.

### The Block System.

Thirdly, we have the so-called "block system," in which the hospital is built as a series of connected blocks, the several units forming the various departments being arranged not in special groups, but in one building. This is the prevailing type of construction adopted in the older hospitals, and was considered to have many disadvantages. Latterly, however, there has arisen a school of opinion that favours the readoption, within limits, of this type. Its advantages are compactness, with subsequent economy of space, and relatively greater ease of administration in comparison with the pavilion type; its main disadvantage is that it tends to cramp the departments unduly, and that it offers great difficulties to the constructor to secure the maximum of air space and ventilation necessary in a modern hospital.

There are, of course, numerous types which are modifications or combinations of these three main ones. Questions of site, cost, nature of work done, and other important problems have to be considered in devising a hospital, and it is not always possible to adopt the plan that, theoretically, is the best. To hospital workers one of the most interesting lessons that can be gleaned from a survey of the world's hospitals is the manner in which architects have compromised on questions of construction, and evolved institutions which are singularly excellent in view of the grave difficulties of construction that confronted the builders. As an instance in point, we may cite the English hospital at Constantinople, and, perhaps, the surgical clinic at Gottingen.

### The Best Type.

Which of these types, then, is to be held as the most suitable for the ward unit in its best sense? This question is not easy to answer, and individual opinion will largely weigh in arriving at a conclusion. In general, however, it may be stated that the unit, as a complete entity, is seen at its best in those hospitals which have adopted the single-pavilion system. In such, each pavilion is a separate and complete entity; each forms a distinct unit, but the department, comprising so many different units, may be scattered. In the compound-pavilion system, the great advantage is that the department is more or less compact. For our purpose, the description of the ideal unit, it does not much matter which type we adopt, though for the sake of convenience we may confine our attention to the single-pavilion system.

